# Cardiac Conduction Delay for Sodium Channel Antagonist Antiseizure Medications: An Analysis of the Canadian Longitudinal Study on Aging

**DOI:** 10.1212/WNL.0000000000210302

**Published:** 2025-02-03

**Authors:** Nathan A. Shlobin, Jimmy Li, Josemir W. Sander, Mark Robert Keezer, Roland D. Thijs

**Affiliations:** 1 Department of Neurosurgery, Neurological Institute of New York, New York Presbyterian Hospital—Columbia University Medical Center, NY;; 2 Stichting Epilepsie Instellingen Nederland (SEIN), Heemstede, the Netherlands;; 3 Department of Neurology, Leiden University Medical Centre, the Netherlands;; 4 Centre Hospitalier de l'Université de Sherbrooke (CHUS), Canada;; 5 Research Centre of the Centre Hospitalier de l'Université de Montréal (CRCHUM), Canada;; 6 UCL Queen Square Institute of Neurology, and Chalfont Centre for Epilepsy, Chalfont St Peter, London, United Kingdom;; 7 Department of Neurology, West China Hospital, Sichuan University, Chengdu, China;; 8 School of Public Health, Université de Montréal, Canada; and; 9 Centre Hospitalier de l'Université de Montréal (CHUM), Canada.

## Abstract

**Background and Objectives:**

People with epilepsy are at risk of cardiac arrhythmias. Whether this association results from epilepsy, antiseizure medications (ASMs) such as sodium channel blockers (NABs), or other factors has not been systematically assessed. The aims of this study were to quantify the odds of cardiac conduction delays (CCDs) on electrocardiogram in older people with active epilepsy using vs not using NABs, to determine the prevalence of CCDs by NABs, and to examine the association of demographic and clinical factors with CCDs.

**Methods:**

This was a cross-sectional study of the Canadian Longitudinal Study on Aging. We defined active epilepsy as self-reported epilepsy and taking ASM. Sodium channel blockers (NABs) were phenytoin, lamotrigine, carbamazepine, oxcarbazepine, or lacosamide. We compared CCDs between people with epilepsy using NABs and those not using NABs; determined the prevalence of CCDs by NAB type; and fitted a logistic regression model for each abnormal ECG outcome as a function of active epilepsy and NAB use while adjusting for demographics and clinical factors. Multiple imputations handled missing data (200 iterations).

**Results:**

In total, 30,077 people, with mean age 63.0 (10.25) years and 50.9% female, were studied, including 141 people with active epilepsy who used NABs, 68 who did not use NABs, and 29,868 who did not have active epilepsy. Demographics between groups and relative to people without epilepsy were similar. People with active epilepsy taking NABs were more likely to have prolonged QRS (odds ratio [OR] = 2.85 [95% CI 1.09–7.43]) and any CCD (1.94 [1.03–3.63]) compared with those with active epilepsy without NAB. After adjusting for Framingham score and heart rate–lowering medications, NAB use was associated with prolonged QTc (OR = 1.52 [95% CI 1.06–2.18]) and any CCD (1.78 [1.16, 2.74]). The prevalence of any CCD was 36.1% [95% CI 24.2%–49.4%] for carbamazepine, 45.5% [31.7%–58.5%] for phenytoin, and 54.7% [28.9%–75.6%] lamotrigine. Epilepsy was not associated with any CCD.

**Discussion:**

People with active epilepsy using NABs more commonly have CCDs. NAB use is associated with CCD, whereas active epilepsy is not.

## Introduction

People with epilepsy are at risk of premature mortality, of which sudden unexpected death in epilepsy and sudden cardiac death (SCD) are primary and partly overlapping entities.^1,2^ Recent work has examined the interface between neurologic and cardiac factors in promoting premature mortality. Such work has led to the concept of the epileptic heart.^3^ Damage to the heart and coronary vasculature from chronic epilepsy may predispose people to electrical and mechanical dysfunction.^4^

Antiseizure medications (ASMs) have been identified as a possible determinant of premature mortality in people with epilepsy for 2 primary reasons. First, enzyme-inducing ASMs lower the threshold for cardiovascular events, most likely by promoting atherosclerosis.^5^ Some enzyme-inducing ASMs (EIASMs), such as carbamazepine, precipitate an increase in lipid levels.^6^ Second, there is interest in examining drugs that may induce arrhythmias, such as sodium channel blockers (NABs).^7^ Variations in genes encoding sodium channels lead to cardiac conduction disease and arrhythmias.^8^ Pharmacologic studies have indicated that NABs slow cardiac conduction.^4^

Addressing the role of ASMs is important because ASM type is a modifiable factor. The extent to which seizures, NAB use, or both together contribute to cardiac conduction delays (CCDs) is unclear. The relationship between NABs and CCDs on ECG in epilepsy has not been previously examined. We compare the odds of CCD on ECG between people with active epilepsy who use NAB and those who do not, determine the prevalence of CCDs by NAB, and assess the association and importance of demographic and clinical factors with CCD.

## Methods

### Database

Our study is a cross-sectional analysis of the Canadian Longitudinal Study on Aging (CLSA), an ongoing prospective study of 51,338 adults aged 45–85 years enrolled in Canada.^9^ We used baseline CLSA data collected between 2011 and 2015. CLSA exclusion criteria at baseline were residing at a long-term care facility, cognitive impairment, being a full-time member of the military, living on a First Nations reserve, and being unable to communicate in English or French.^9^ The CLSA Comprehensive cohort, including approximately 30,000 individuals, was thoroughly evaluated at one of 11 data collection centers in 7 of 10 Canadian provinces. Eligibility for the Comprehensive cohort required living within 25–50 km of one such center.^9^ Our study focuses on the Comprehensive cohort, given that this cohort collects data from clinical/physical tests and biospecimens.^9^ These tests included a resting 12-lead ECG (GE MAC 1600) and a 10-second recording, from which quantitative measurements of the PQ interval, QRS length, and QTc were extracted automatically.

### Variables

Our primary outcomes were ECG conduction delays. We classified abnormal intervals rather than analyze continuous data to remove the influence of potential outliers. Long PQ was defined as >200 ms per the 2018 American College of Cardiology/American Heart Association/Heart Rhythm Society Guidelines on evaluating and managing bradycardia and CCD.^10^ This definition of long PQ is based on a community-based cohort study that found that PQ > 200 ms was associated with a higher incidence of pacemaker implantation and all-cause mortality.^11^ We chose lower categories for QRS and QTc length than the 2018 guideline because of their association with cardiac disease and mortality. A study combining data from 3 population-based cohorts from different periods determined that QRS duration was associated with a significantly increased risk of SCD with a pooled hazard ratio of 1.030 per 1-ms increase, and QTc interval had a borderline to significant association with SCD with a pooled hazard ratio of 1.007 per 1-ms increase.^12^ Long QRS was >100 ms given the association between this cutoff and ventricular dysfunction^12–15^ in large hospital-based and population-based studies. Long QTc was >430 ms for men and >450 ms for women because these are the cutoff values traditionally used for borderline QTc prolongation^16,17^ and associated with increased mortality risk.^18,19^ A population-based study found QTc ≥430 ms was associated with decreased survival with a relative risk of 2.4, regardless of sex.^20^ The study combining 3 population-based cohorts also indicated that a QTc ≥428.8 was associated with increased SCD risk.^12^ CCD was a composite outcome denoting whether an individual had any one of these delays. Of note, a single individual could have multiple CCDs.

Our exposure of interest was active epilepsy. A previously developed diagnostic algorithm using the CLSA to identify individuals with active epilepsy was considered. This algorithm uses self-reported information on epilepsy diagnosis, ASM use, and epilepsy-related symptoms to identify people with active epilepsy (sensitivity >99%, specificity >98%).^21^ Applying this algorithm directly to baseline data was not feasible given its introduction with the first 3-year follow-up (conducted between 2015 and 2018). We defined individuals with active epilepsy as either (1) those with a positive algorithm screen at the first follow-up and who took an ASM at baseline or (2) those at baseline who reported having a history of epilepsy (a single self-report question, “Has a doctor ever told you that you have epilepsy?”) and took an ASM. Some people who reported having epilepsy at the follow-up provided their age at seizure onset. If they reported developing epilepsy within the past 3 years (i.e., between the baseline and first follow-up), their screen was considered negative.

Other variables of interest (all measured at baseline) included age, sex, ethnicity, household income, level of education, Framingham cardiovascular risk score,^22^ ASM use, NAB use, and heart rate–lowering medication use. NABs were phenytoin, lamotrigine, carbamazepine, oxcarbazepine, and lacosamide. No individuals in the sample used eslicarbazepine, rufinamide, or felbamate. Heart rate–lowering medications were β-blockers and calcium channel blockers.

### Statistical Analyses

We described the general characteristics of our cohort stratified by active epilepsy status and NAB use. We compared CCDs between people with epilepsy using NABs and those not using NAB, reporting unadjusted odds ratios with 95% CIs. The prevalence of CCDs by NAB was also determined.

We fitted a logistic regression model for each abnormal ECG outcome as a function of active epilepsy, NAB use, age, and sex (model A). We fitted additional models adjusting for using heart rate–lowering medications (model B), Framingham cardiovascular risk score (model C), and both variables at the same time (model D). Estimates from these models were pooled using Rubin rules,^23^ from which ORs with 95% CIs were extracted. We weighted our models using the analytical sampling weights provided by the CLSA designed to correct for the CLSA stratified sampling strategy and nonresponse, rendering results representative of the Canadian population.^24^

Missing data were handled with multiple imputations (200 iterations) using the *mice* R package.^25^ Reported means and counts represent the averages across all imputations. As such, count data could take on noninteger values. Appropriate convergence of the multiple imputation model can be observed in eFigure 1. We repeated our primary descriptive analyses using nonimputed data (complete cases) as sensitivity analyses.

We used STATA 17 for descriptive statistics and R for comparative and multiple imputations. Continuous variables are presented as mean (SD). Categorical variables are presented as count (proportion). Statistical significance was defined as an α level of <0.05.

### Ethical Approval

Thirteen ethics boards across the Canadian provinces approved the CLSA. All participants provided written informed consent. The Research Ethics Board of the Centre Hospitalier de l'Université de Montréal ('#21.355) approved this analysis of the CLSA data.

### Data Availability

Data are available from the CLSA (clsa-elcv.ca) for researchers who meet the criteria for access to deidentified CLSA data.

## Results

### Demographics

**Figure 1:**
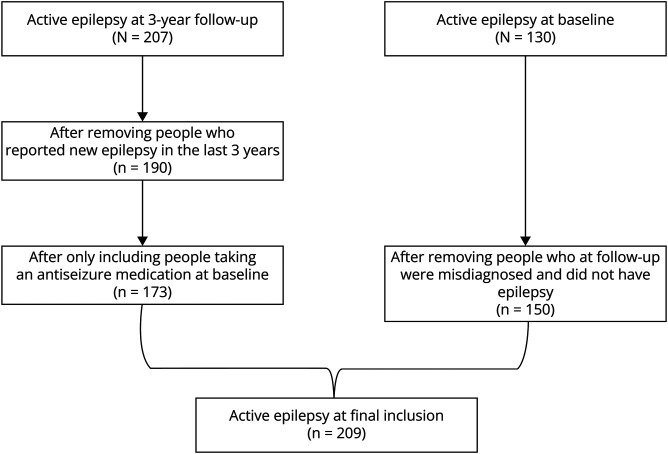
Flowchart for Selection of the Active Epilepsy Cohort

Figure 1 presents a flowchart of this study. Of 30,077 participants in the Comprehensive cohort, 29,868 did not have active epilepsy (AE), 141 had active epilepsy and used NAB (+AE/+NAB), and 68 had active epilepsy but did not use NAB (+AE/–NAB). Table 1 lists similar cohort characteristics between the data sets. The mean age was 63.0 (10.25) years, and 15,320 participants (50.9%) were female. ASM use was noted in 1,160 (3.9%) individuals, NAB ASM in 258 (0.9%), and heart rate–lowering medications in 3,526 (11.7%). The demographics were generally similar between the groups. An exception was that the proportion of women was lower in the +AE/+NAB group than in the +AE/-NAB group (36.8% vs 58.5%, respectively). The same data sets are presented in eTable 1, without replacement of the missing data, with generally similar results to the data sets after multiple imputations.

**Table 1:** Cohort Characteristics, Stratified by Use of Sodium Channel Blockers^a^

	Active epilepsy and using NAB (n = 141)	Active epilepsy and not using NAB (n = 68)	No active epilepsy (n = 29,868)	Participants with missing data
Mean age (SD), y	62.1 (10.0)	60.6 (10.3)	63.0 (10.3)	0 (0)
Female, n (%)	52.0 (36.8)	40.0 (58.5)	15,228.0 (51.0)	0 (0)
Ethnicity, n (%)				
White	136.2 (96.5)	63.2 (92.4)	28,207.0 (94.4)	32 (0.1)
Asian	2.0 (1.4)	2.0 (2.9)	714.6 (2.4)	
Black	0.0 (0)	1.0 (1.5)	262.6 (0.9)	
Latin American	0.0 (0)	0.2 (0.3)	125.8 (0.4)	
Arab	0.0 (0)	0.0 (0)	104.0 (0.4)	
Other	3.0 (2.1)	2.0 (2.9)	473.4 (1.6)	
Household income, n (%)				
< $20,000	23.0 (16.3)	12.4 (18.1)	1,678.8 (5.6)	1,941 (6.5)
$20,000-$50,000	30.0 (21.3)	20.2 (29.5)	6,840.2 (22.9)	
$50,000-$100,000	55.8 (39.5)	17.4 (25.4)	10,523.8 (35.2)	
$100,000-$150,000	19.0 (13.5)	10.4 (15.2)	5,831.0 (19.5)	
> $150,000	13.4 (9.5)	8.0 (11.7)	5,013.6 (16.8)	
Level of education, n (%)				
No postsecondary degree	21.6 (15.3)	13.8 (20.2)	3,810.4 (12.8)	4,545 (15.1)
Nonuniversity postsecondary degree	46.2 (32.7)	28.2 (41.2)	10,205.3 (34.2)	
Undergraduate degree	37.6 (26.6)	13.0 (19.1)	9,131.8 (30.6)	
Postgraduate degree	35.8 (25.4)	13.4 (19.6)	6,739.8 (22.6)	
Framingham score, mean (SD)	12.3 (5.1)	12.4 (4.4)	12.6 (4.7)	3,576 (11.9)
Use of ASMs, n (%)	141.2 (100)	68 (99.4)	950.8 (3.2)	0 (0)
Use of heart rate–lowering medications, n (%)	16.2 (11.5)	6.0 (8.8)	3,507.8 (11.7)	0 (0)
History of stroke, self-reported, n (%)	18.2 (12.9)	5.0 (7.3)	502.8 (1.7)	121 (0.4)
History of transient ischemic attack, self-reported, n (%)	11.2 (7.9)	4.0 (5.8)	965.8 (3.2)	242 (0.8)
History of myocardial infarction, self-reported, n (%)	6.6 (4.7)	6.0 (8.8)	1,463.4 (4.9)	149 (0.5)

Abbreviations: ASMs = antiseizure medications; NAB = Na channel blocker.

a Missing data replaced with multiple imputation, which leads to noninteger counts. All cells are n (%), unless reported otherwise.

### CCDs

**Figure 2:**
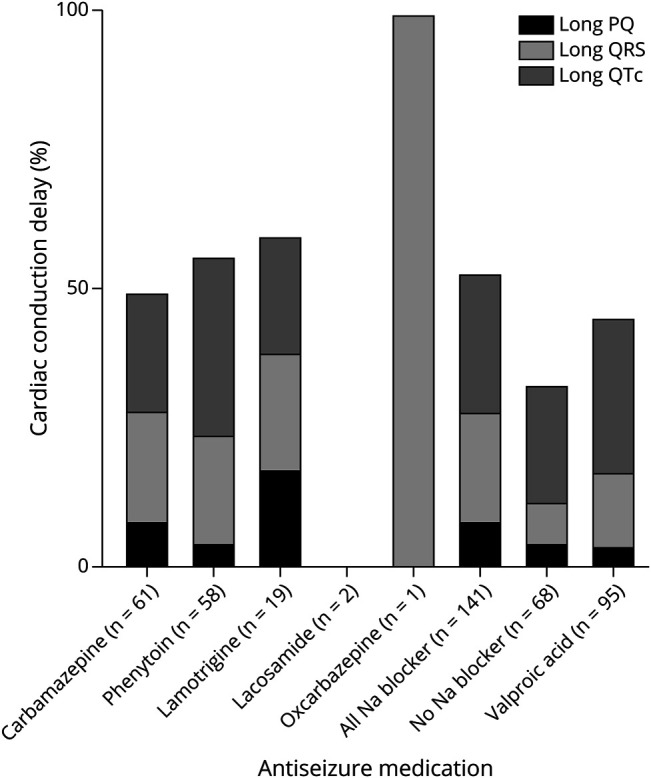
Percentages of Types of Cardiac Conduction Delays by Sodium Channel Blocker

Table 2 reports comparisons of CCDs on ECG between the +AE/+NAB and +AE/–NAB groups. The +AE/+NAB group had a greater prevalence of prolonged QRS (OR = 2.85 [95% CI 1.09–7.43]) and any CCD (1.94 [1.03–3.63]) but not prolonged PQ or QTc. eTable 2 presents similar results for the complete case data sets. Table 3 presents CCDs by specific NAB. Figure 2 presents the percentages of types of CCDs by NAB. The prevalence of any CCD was 36.1%–54.7% for the most commonly used NABs.

**Table 2:** Cardiac Conduction Delays on ECG Between People With Active Epilepsy Using Sodium Channel Blockers and People With Active Epilepsy Who Did Not Use Sodium Channel Blockers^a^

	Active epilepsy and using NAB (n = 141)	Active epilepsy and not using NAB (n = 68)	Unadjusted OR for cardiac conduction delay between active epilepsy and using NAB and active epilepsy and not using NAB, (95% CI)
Prolonged PQ interval	11.2 (7.9)	3.0 (4.4)	1.88 (0.51–6.97)
Prolonged QRS duration	28.6 (20.3)	5.6 (8.2)	**2.85 (1.09–7.43)**
Prolonged QTc interval	35.4 (25.1)	14.6 (21.4)	1.23 (0.62–2.47)
Any cardiac conduction delay	59.8 (42.4)	18.8 (27.5)	**1.94 (1.03–3.63)**

Abbreviation: NAB = Na channel blocker; OR = odds ratio.

a All cells are n (%), unless reported otherwise. Any cardiac conduction delay tabulates the people who had at least 1 cardiac conduction delay on ECG.

**Table 3:** Cardiac Conduction Delays by Type of Sodium Channel Blocking Antiseizure Medication^a^

	Prolonged PQ interval	Prolonged QRS duration	Prolonged QTc interval	Cardiac conduction delay on electrocardiogram
Carbamazepine (n = 61)	8.5 (2.7–18.1)	20.0 (10.6–31.8)	21.0 (11.9–33.7)	36.1 (24.2–49.4)
Phenytoin (n = 58)	4.5 (1.1–14.4)	19.7 (9.9–31.4)	32.1 (21.0–46.3)	45.5 (31.7–58.5)
Lamotrigine (n = 19)	17.9 (3.4–39.6)	21.1 (6.1–45.6)	21.1 (6.1–45.6)	54.7 (28.9–75.6)
Lacosamide (n = 2)	0 (0–84.2)	0 (0–84.2)	0 (0–84.2)	0 (0–84.2)
Oxcarbazepine (n = 1)	0 (0–97.5)	100 (2.5–100)	0 (0–97.5)	100 (2.5–100)
All NAB (n = 141)	7.9 (4.0–13.5)	20.3 (14.2–28.2)	25.1 (17.9–32.8)	42.4 (34.3–51.2)
No NAB (n = 68)	4.4 (0.9–12.4)	7.7 (2.4–16.3)	21.2 (11.7–32.2)	27.1 (16.5–38.6)

Abbreviation: NAB = Na channel blocker.

a All cells are % (95% CI).

### Adjusted Analyses

Table 4 provides the output of logistic regression analyses by type of CCD. Model D indicated that NAB was associated with increased odds of prolonged QTc (OR = 1.52 [95% CI 1.06, 2.18]) and any CCD (1.78 [1.16, 2.74]) after adjusting for age, sex, Framingham score, and the presence of heart rate–lowering medications. Active epilepsy was not associated with CCD. Models A, B, and C generated similar results.

**Table 4:** Cardiac Conduction Delays by Sodium Channel Blocker, Adjusted for Possible Confounding

	Prolonged PQ interval, OR (95% CI)	Prolonged QRS duration, OR (95% CI)	Prolonged QTc interval, OR (95% CI)	Any cardiac conduction delay on electrocardiogram, OR (95% CI)
Model A: Heart rate–lowering medication and Framingham score not included				
Age	1.07 (1.07–1.08)	1.04 (1.04–1.05)	1.03 (1.03–1.03)	1.04 (1.04–1.04)
Sex	0.44 (0.39–0.49)	0.24 (0.22–0.26)	0.38 (0.36–0.40)	0.32 (0.30–0.34)
Active epilepsy	0.80 (0.24–2.71)	1.12 (0.64–1.94)	0.72 (0.45–1.15)	0.78 (0.50–1.22)
Na channel blocker	1.29 (0.45–3.69)	1.44 (0.84–2.46)	1.60 (1.11–2.30)	1.85 (1.21–2.83)
Model B: Heart rate–lowering medication included				
Age	1.06 (1.06–1.07)	1.03 (1.03–1.04)	1.03 (1.03–1.03)	1.03 (1.03–1.04)
Sex	0.46 (0.41–0.51)	0.25 (0.23–0.27)	0.39 (0.36–0.41)	0.32 (0.31–0.34)
Active epilepsy	0.79 (0.23–2.72)	1.11 (0.63–1.94)	0.72 (0.45–1.15)	0.78 (0.49–1.22)
Na channel blocker	1.28 (0.45–3.70)	1.44 (0.84–2.46)	1.60 (1.11–2.30)	1.84 (1.20–2.83)
Heart rate–lowering medication	2.05 (1.75–2.41)	1.73 (1.55–1.94)	1.34 (1.22–1.47)	1.51 (1.38–1.67)
Model C: Framingham score included				
Age	1.07 (1.06–1.08)	1.04 (1.02–1.06)	1.00 (1.00–1.00)	1.01 (1.01–1.02)
Sex	0.45 (0.39–0.50)	0.27 (0.24–0.30)	0.49 (0.46–0.53)	0.39 (0.37–0.42)
Active epilepsy	0.80 (0.24–2.71)	1.14 (0.66–1.99)	0.76 (0.47–1.20)	0.81 (0.51–1.27)
Na channel blocker	1.29 (0.45–3.67)	1.40 (0.82–2.38)	1.52 (1.06–2.18)	1.78 (1.16–2.74)
Framingham score	1.01 (0.99–1.03)	1.04 (1.02–1.06)	1.10 (1.09–1.11)	1.08 (1.07–1.09)
Model D: Heart rate–lowering medications and Framingham score included				
Age	1.07 (1.06–1.08)	1.02 (1.02–1.03)	1.00 (1.00–1.00)	1.01 (1.01–1.02)
Sex	0.45 (0.39–0.50)	0.27 (0.24–0.30)	0.49 (0.46–0.53)	0.39 (0.37–0.41)
Active epilepsy	0.79 (0.23–2.69)	1.13 (0.65–1.97)	0.75 (0.47–1.20)	0.80 (0.51–1.27)
Na channel blocker	1.29 (0.44–3.74)	1.41 (0.82–2.40)	1.52 (1.06–2.18)	1.78 (1.16–2.74)
Framingham score	0.99 (0.97–1.01)	1.03 (1.01–1.05)	1.10 (1.09–1.11)	1.08 (1.06–1.08)
Heart rate–lowering medication	2.08 (1.77–2.45)	1.66 (1.49–1.85)	1.17 (1.06–1.29)	1.37 (1.24–1.50)

Abbreviation: OR = odds ratio.

## Discussion

We compared the prevalence of CCD on ECG between people with active epilepsy using NAB or not on a baseline ECG. We also characterized the CCD types across various NABs. Finally, we examined the association and importance of demographic and clinical factors with CCD. Our findings suggest that NAB use, rather than active epilepsy, is associated with CCD.

The overlap between epilepsy and cardiovascular disease (CVD) has been characterized for several outcomes, including the increased risk of sudden cardiac arrest,^26^ arrhythmias, ischemic events, and potential genetic risk factors.^2^ Characteristics of epilepsy, other comorbidities, and ASMs influence the occurrence of cardiovascular events. ASMs have drawn interest as clinicians may modify ASM regimens.^27^ A population-based study in Wales reported that people with epilepsy on ASM treatment have a greater risk of major cardiovascular events than population-based controls.^28^ A Finnish prospective case-control study found a higher rate of SCD among ASM users.^5^ An English population-based study suggested a dose-dependent increased hazard of incident CVD for people using EIASMs.^29^ The Welsh study and a Japanese retrospective cohort study found no such association.^28,30^ A retrospective case-control cohort reported that carbamazepine and valproic acid are associated with increased risk of cardiovascular events relative to lamotrigine.^31^ ASMs also influence the risk of arrhythmias. Carbamazepine and valproic acid (VPA) are associated with an increased risk of arrhythmias such as atrial fibrillation.^32^ Otherwise, existing evidence has been equivocal. An administrative claims database study using propensity score weighting highlighted that the combined end point of SCD and ventricular arrhythmia was less frequent in adults with newly prescribed levetiracetam than those prescribed oxcarbazepine.^33^ A systematic review indicated that lacosamide users had a significantly higher risk of arrhythmias, whereas those using levetiracetam did not.^34^ Another found insufficient evidence to support or refute an association between lamotrigine and sudden death or ECG changes.^35^ A strong focus on the sodium channel is warranted because of the role of the sodium channel variations and NABs in promoting CCDs and arrhythmias.^8^

Investigators have examined interictal ECGs to determine whether baseline CCDs yield insight into who are at risk of future arrhythmias and sudden death. There have been suggestions of CCD in people with epilepsy in small clinical cohorts, mainly focusing on refractory epilepsy.^4^ There is, however, no existing systematic investigation of CCD in a large sample, and no such investigation has occurred in a standardized manner concerning a drug class. We add to the evidence by providing a focused examination of the CCDs in people with epilepsy with an emphasis on NABs. Ictal events can aggravate CCDs, perhaps precipitating arrhythmias.^4^

Our findings indicate that over one-third of people with active epilepsy have a CCD on baseline ECG. A recent Food and Drug Administration safety warning regarding the cardiac effects of lamotrigine prompted an International League Against Epilepsy/American Epilepsy Society (AES) Task Force to recommend consideration of an ECG in people older than 60 years who will be initiating lamotrigine and in people younger than 60 with known cardiac disease or risk factors of CVD.^36^ The high proportion of any CCD in people using and not using NAB indicates that acquiring an ECG may be worthwhile in people with epilepsy using ASMs who use NABs and are 60 years or older or who have risk factors of arrhythmia.

Active epilepsy was not associated with any CCD, which would appear at odds with previous studies.^37,38^ Possibly, epilepsy in and of itself does not predispose people to CCDs. Other studies may have missed this because of a lack of adjustment for confounding or separation of people taking NABs vs not taking NABs. Some people with active epilepsy have reduced conduction intervals, including shorter QRS and QTc intervals, indicative of repolarization abnormalities.^39^ Our definition of epilepsy requires the use of ASMs. Some ASMs, such as primidone and potentially phenytoin or carbamazepine, may lead to QTc shortening in some people with epilepsy.^40^ Our analysis was restricted to the prevalence of CCD and did not explore whether some ASMs including NAB could shorten conduction intervals. Chronic epilepsy has been associated with reduced vagal tone as manifested by reduced root mean square of successive differences in heart rate variability.^41^ It is possible that reduced heart rate variability secondary to epilepsy may have accounted for the lack of association between epilepsy and conduction delay.

We found a higher proportion of QRS prolongation and any-CCD people with active epilepsy who used NAB. Our regression analyses indicated that NAB use was associated with any CCD and QTc prolongation. The discrepancy between the significance of QRS but not QTc interval on univariable analysis and of QTc but not QRS interval on multivariable analysis may reflect the need to account for the effect of heart rate–lowering medications. Heart rate–lowering medication use was associated with prolonged PQ, QRS, QTc, and any CCD. These associations vary by medication and agent, but calcium channel blockers prolong PQ interval.^42,43^ β-Blockers generally increase PQ interval^44^ and may shorten or lengthen QTc based on heart rate.^45^

Over one-third of people with one of the 3 most commonly used NABs (carbamazepine, phenytoin, and lamotrigine) had any CCD. Prolonged QRS intervals occurred in one-fifth of these same individuals. Prolonged QTc intervals occurred in over one-fifth, and nearly one-fifth of lamotrigine users had PQ prolongation. These findings support the hypothesis that NAB has a class effect in prolonging cardiac conduction. Previously, class effects for ASMs have generally focused on EIASMs.^29^ We did not classify VPA as a NAB because its mechanism of action is poorly understood, and sodium channel blockade is unlikely to contribute to its antiseizure effects.^46^ Nevertheless, valproic acid acts on sodium channels in the brain^47^ and heart.^48^ Our findings suggest that over one-third of VPA users had any CCD, including over one-quarter with a prolonged QTc interval, supporting its potential role in the development of cardiac arrhythmia.^32^

Our study has strengths. The CLSA population-based nature allows results to be generalizable. We identified CCDs by type of NAB. The CLSA database includes standardized ECG for all participants in the Comprehensive cohort, increasing their quality. Constructing multiple regression models allowed us to disentangle the independent associations of active epilepsy, NAB, cardiovascular risk, and heart rate–lowering medication on CCDs. Multiple imputation provided a rigorous method to handle missing data.

Our study has limitations. First, we used a modified version of an epilepsy diagnostic algorithm at baseline. The original algorithm had excellent diagnostic accuracy,^21^ but this does not apply to the modified algorithm. Misclassification from the algorithm would likely have biased results toward the null, underestimating the strength of the association between NAB and CCD. Second, we could not determine the full range of cardiovascular comorbidities, with a history of stroke, TIA, and myocardial infarction only available from self-reported data. We included use of heart rate–lowering medications and the Framingham cardiovascular risk score in our regression models to account for baseline cardiovascular risk. Third, multiple imputations are based on the assumption of missing at random. If data were not randomly missing, the results would be biased. Our sensitivity analyses suggest that the imputed data did not differ appreciably from complete case data. Fourth, ECG parameters in the CLSA were automatically estimated by recorders. Automatically estimated ECG parameters differ across manufacturers and provide variable results relative to human rater assessment.^49,50^ The ECGs performed the data collection sites followed a written protocol using the same machine type and brand. Fifth, there is often debate regarding what constitutes an abnormal interval on ECG, particularly given these intervals vary by age and sex.^51^ We used commonly accepted thresholds for adults and accounted for sex differences in QTc intervals. We used our borderline cutoffs for prolonged QRS and QTc abnormalities to ensure that CCDs associated with cardiac dysfunction and mortality were accounted for. The sample sizes of people with active epilepsy who used certain NABs were small (e.g., lacosamide), leading to some imprecision in certain results. Additional repolarization properties, such as QT dispersion, have been linked to sudden arrhythmic death in the general population. We could not confirm the increased prevalence of QT dispersion in at least one-third of people with chronic epilepsy^3^ because the CLSA only reports conduction intervals.

We found that people with epilepsy using NABs are more likely to have a CCD. Sodium channel blocker use, not epilepsy, is most associated with CCD. Age, sex, Framingham score, and heart rate–lowering medication use are also associated with CCD. The proportion of CCD types differs by NAB. Still, our analysis indicates that over half of lamotrigine users experience a CCD, slightly under half for phenytoin, and over one-third for carbamazepine. This suggests that clinicians should exercise caution when prescribing NAB to people who are predisposed to cardiac arrhythmias. An ECG may be warranted for people with epilepsy and treated with an NAB, mainly if they are aged at least 60 years or have risk factors of arrhythmia.

## Glossary

ASM: antiseizure medication
CCD: cardiac conduction delay
CIHR: Canadian Institutes of Health Research
CLSA: Canadian Longitudinal Study on Aging
CVD: cardiovascular disease
EIASM: enzyme-inducing ASM
NAB: sodium channel blocker
OR: odds ratio
SCD: sudden cardiac death
